# Ultra-stable nano-micro bubbles in a biocompatible medium for safe delivery of anti-cancer drugs

**DOI:** 10.1038/s41598-024-55654-w

**Published:** 2024-03-04

**Authors:** Ulviye Bunyatova, Mustafa Dogan, Engincan Tekin, Onur Ferhanoğlu

**Affiliations:** 1 Biomedical Engineering Department, Engineering Facility, Baskent University, Ankara, Turkey; 2 Department of Control and Automation Engineering, Faculty of Electrical-Electronics Engineering, Istanbul Technical University, Istanbul, Turkey; 3 Department of Electronics and Communications Engineering, Faculty of Electrical-Electronics Engineering, Istanbul Technical University, Istanbul, Turkey

**Keywords:** Ultra-stable sub-millimeter bubbles AgNPs colloidal, Photosensitive bio-nanocomposite, Bubble modeling, Multi-layered structure, Low laser excitation, Nanoparticles, Applied mathematics, Optical manipulation and tweezers

## Abstract

We conducted a series of experimental investigations to generate laser-stimulated millimeter bubbles (MBs) around silver nanoparticles (AgNPs) and thoroughly examined the mechanism of bubble formation within this nanocomposite system. One crucial aspect we explored was the lifetime and kinetics of these bubbles, given that bubbles generated by plasmonic nanoparticles are known to be transient with short durations. Surprisingly, our findings revealed that the achieved lifetime of these MBs extended beyond seven days. This impressive longevity far surpasses what has been reported in the existing literature. Further analysis of the experimental data uncovered a significant correlation between bubble volume and its lifetime. Smaller bubbles demonstrated longer lifetimes compared to larger ones, which provided valuable insights for future applications. The experimental results not only confirmed the validity of our model and simulations but also highlighted essential characteristics, including extended lifetime, matching absorption coefficients, adherence to physical boundary conditions, and agreement with simulated system parameters. Notably, we generated these MBs around functionalized AgNPs in a biocompatible nanocomposite medium by utilizing low-power light excitation. By readily binding potent cancer drugs to AgNPs through simple physical mixing, these medications can be securely encapsulated within bubbles and precisely guided to targeted locations within the human body. This capability to deliver drugs directly to the tumor site, while minimizing contact with healthy tissues, can lead to improved treatment outcomes and reduced side effects, significantly enhancing the quality of life for cancer patients.

## Introduction

A drug delivery system (DDS) employs a variety of techniques to deliver the required quantity of medications to the required location within the required time, boost utilization effectiveness, and lessen unwanted effects^1^. When compared to traditional DDS, stimuli-responsive smart DDS that can react to both endogenous and external stimuli offer special benefits and promising futures for lowering drug toxicity, minimizing drug concentration variations, and enhancing therapeutic efficacy^2,3^. Bubbles are significant medication delivery carriers and they have been discovered with the quick development of biomedical technology. These bubbles span a range of sizes, starting from nanoscale nucleation and progressing to an equilibrium state where they exhibit diameters within the micro and millimeter ranges. As innovative therapeutics continue to emerge, a particular treatment method involving nanocarriers and bubbles is gaining significant attention^4^. Bubbles are evolving as crucial contrast agents for imaging and medication delivery vehicles to specific regions due to their following benefits^5–8^: (1) Low density and high deformability. (2) The bubble core's compressible structure makes it easier for genes to marginate and for extravasation through the extracellular space to be boosted. (3) using targeted microbubbles to administer toxic cancer drugs, e.g. chemotherapy drugs. Silver nanoparticles (AgNPs) due to distinct physical–chemical characteristics and biological activity in comparison to bulk materials, are the focus of much research^9–16^. Functionalized AgNPs encapsulated within bubbles serve as an effective anticancer agent on their own and also function as safe and durable vehicles for delivering chemotherapy drugs to the target area without side effects^17–19^. Extensive research has explored the potential of nanoparticles for targeted gene and drug therapy^20^. Recent studies show that AgNPs can be used as nanocarriers for desired medications to cure cancer. Wang et al.^21^ synthesized Folic acid (FA)-coated AgNPs and successfully conjugated them with the chemotherapeutic drug doxorubicin (DOX) using electrostatic bonding. Following an efficient 8-h release of DOX, a significant and visible cell death was observed, demonstrating the promising potential of this approach for targeted cancer therapy. In an effort to enhance intracellular uptake and cytotoxicity in lymphoma cells, Fang et al.^22^ using AgNPs developed self-assembled polymer-doxorubicin complexes, employing functional groups such as imidazole and tertiary amine, along with three distinct cationic side chains. Locatelli et al.^23^. successfully developed a nanocarrier by entrapping lipophilic AgNPs within PEG-based polymeric nanoparticles that also contained chlorotoxin. Furthermore recent studies have revealed that AgNPs possess not only nanocarrier functions but also exhibit a high degree of plasmon tunability, making them the most prominent nanoscale product among various related nanoparticles^24^. This unique characteristic of AgNPs is harnessed in hyperthermia procedures, where they are utilized to target and remove solid tumors from specific regions using radiation^25–28^.

However, the above-described protocols for chemo and radiation therapy have limitations due to unexpected drug-associated side effects, a lack of specificity of low drug concentrations at the tumor target site, and the development of chemoresistance^29,30^.

Overcoming the challenges associated with safe drug delivery products and their translation from preclinical to clinical applications can be achieved through innovative nanoparticle-stabilized bubbles engineering approach.

By initiating bubble nucleation around nanosized functionalized AgNPs within a biocompatible colloidal medium, a robust and persistent delivery vehicle is established to reach precise target areas for cancer treatment. This innovative method minimizes potential side effects while maximizing the effectiveness of the treatment. Moreover, the functionalized AgNP itself exhibits anticancer properties^10,13,14^. For safe drug delivery, a potent chemotherapy drug can be initially attached to the functionalized AgNP through both physical and chemical conjugation methods^22,31–33^. Following this, the entire construct can be integrated within the expanding bubble, establishing a secure and enduring vehicle for delivering chemotherapy drugs to specific target areas. This significant advancement holds the potential to create new opportunities for nanomedicine products within the clinical settings and aims to minimize potential side effects and maximize the efficacy of the treatment. Recently has shown for the first time the sound waves, or ‘acoustical tweezers’, can manipulate and move microbubbles using ultrasound, can trigger the deployment of drugs from the microbubbles, and can do so in a localized way which means they can target specific body parts^34^. This study suggest that acoustical tweezers could be even better than optical ones at manipulating microbubbles, as sound waves can reach deeper into the body.

It is known bubbles generated by plasmonic NPs identified as transient bubbles with short lifetime^35–37^. The majority of optical bubble generation techniques and trapping geometries are reliant on pulsed laser illumination^38,39^. This laser regime induces a robust, momentary temperature surge around illuminated nanoparticles, leading to short-lived bubble nucleation^38,40^. These effects can manifest as energy release in the form of luminescence and/or optical cavitation, triggering local destruction of surrounding species^38,41^ or initiating the generation of shock waves for applications in photoacoustic imaging^42^.The Continuous Wave (CW) method potentially offers greater versatility than pulsed technology, and may enable the creation of a long-lasting bubbles without steady excitation regime. This approach introduces a significant dimension to research in precise sorting, particle manipulation, drug delivery and other biomedical studies. Studies investigating bubbles generated around colloidal nanoparticles under CW laser beams have emerged in literature since beginning 2010, presenting a novel opportunity for utilizing light-matter interaction at the solid–liquid boundary. Authors have published works aiming to theoretically describe bubble generation around metal nanoparticles under continuous wave illumination^43^. Furthermore, recent research by Angelskiy et al.^37,44^ demonstrated the controlled generation of microbubbles through absorptive colloids in water. However, the microbubbles, as observed by these groups, can sustain lifetimes extending to tens of minutes^37^, but exist for extended periods only under specific energy 'feeding' regimes (0.3 W) and do not persist without sustained energy input. Conversely, these bubbles rapidly collapse when the laser power decreases below 0.1 W (A—2018)^44^.

Previous related studies exist in the available literature regarding the appearance of millimeter-sized bubbles. Notably, reports indicate that pulsed laser-induced cavitation bubbles can expand to several millimeters in diameter and endure only for several hundred microseconds, as experimentally investigated using a high-speed camera^45^; Further insights on the similar phenomenon were also detailed in^46^.

Here, we introduce a novel approach aimed at addressing the challenges associated with achieving long-lasting lifetimes of bubbles formed in colloidal solutions through CW laser excitation for short periods.

We conducted a new experimental method for sub-millimeter bubble generation, in which CW is applied to a biocompatible liquid medium containing plasmonic silver nanoparticles (AgNPs). We examined the millimeter – scaled bubble formation mechanism, life-time and kinetics analyses inside of the ultra-diluted biocompatible aqueous nanocomposite system. For this implementation a custom laser set-up was designed. Achieved life-time is over seven days by ending the experiment intentionally. This longevity is much over the reported in literature and beyond expectation. Furthermore, we have relatively low total power due to short–limited excitation times.

## Experimental set-up for bubble forming

This section summarizes the optical methodology in observing laser induced long-life time bubbles.

**Figure 1:**
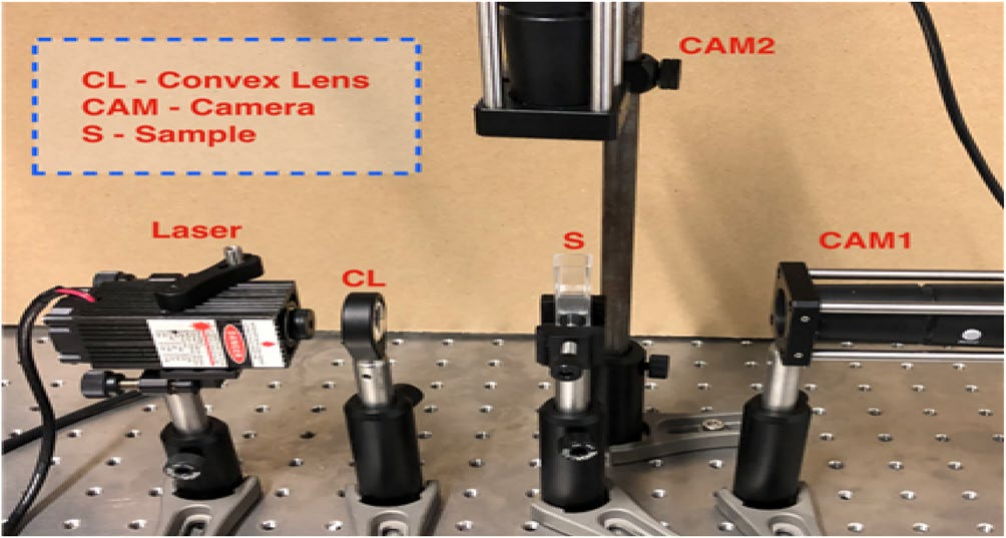
Experimental setup that is used in inducing and observing bubbles within the nanoparticle solution.

Set-up: The custom-made setup, illustrated in Fig. 1 comprises a laser diode having 1Watt optical power and λ = 405 nm wavelength, with an attached lens adjuster. The laser beam was directed to the nanoparticle solution filled cuvette. The 1/e^2^ beam diameter was set to 4 mm on the cuvette and the nanoparticle solution was exposed for 4, 5, and 6 min, corresponding to a total energy of 240, 300, and 360 Joules of optical power. A total of 6 experiments were conducted, where two experiments were conducted per each energy of exposure. The bubble sizes were observed using two cameras replaced at the side and top of the cuvette for a total duration of seven days after the exposures.

## Synthesis and characterization of AgNP in hybrid aqueous nanocomposite

### Synthesis functionalized AgNP in hybrid bicompatible nanocomposite

Aqueous solution of Carboxymethyl cellulose sodium salt (CMC), amine-containing surfactant (octadecylamine(ODA) and light sensitive poly(acrylic acid-co-maleic acid) (MA) were attended as a matrix colloidal, and silver nitrate (AgNO3) salt was used as a metal. Briefly, 0,8 mL of 10% aqueous CMC & 0.05% ODA_ suspension and 0,2 mL of 20% of an aqueous solution of MA were mixed and then 10 mg of silver nitrate (AgNO3) was added and stirred at room temperature for 1 h.

Afterward, nanocomposite was subjected blue LED light irradiation for 15–20 min at room temperature. Actually CMC based nanocomposite possesses carboxyl, primary, and secondary hydroxyl groups on its backbones, providing significant potential for reducing metal nanoparticles (NPs). Additionally, the aqueous blend containing CMC exhibits a generous free space between cross-linked networks during the swollen stage, creating favorable conditions for the nucleation and growth of silver nanoparticles (AgNPs)^47^. The 4.04 eV energy of blue LED is enough to reduces of AgNPs in engineered biocompatible nanocomposite up to size range 4–6 nm in 12 min with functionalized hydroxyl, carboxyl and amide bone sides^12^.

It's important to note that CMC and its derivatives are categorized under natural polysaccharides, known for their non-toxic, highly biocompatible, and biodegradable properties^48,49^. ODA is recognized as an environmentally friendly organic surfactant^50,51^. MA is a hydrophilic, biocompatible, and biodegradable polymer extensively used as a surface-coating ligand for various types of metal nanoparticles^52,53^.The MBs surface was constructed using this biocompatible nanocomposite. Considering the aforementioned referenced literature, the term "biocompatible medium" was used in the manuscript to describe this specific composition.

The interaction between positively charged silver ions and the negatively charged OH and COOH functional groups (OH (hydroxyl)…Ag + and MA−COO−…+Ag) during blue Led treatment occurs through Coulomb electrostatic forces, facilitating selective one-to-one nucleation within the cross-linked nanostructure. Moreover, considering the presence of Van der Waals forces as short-range forces, we can detect the existence of these physical–chemical interactions before the incident light interacts with the nanocomposite blend.

**Figure 2:**
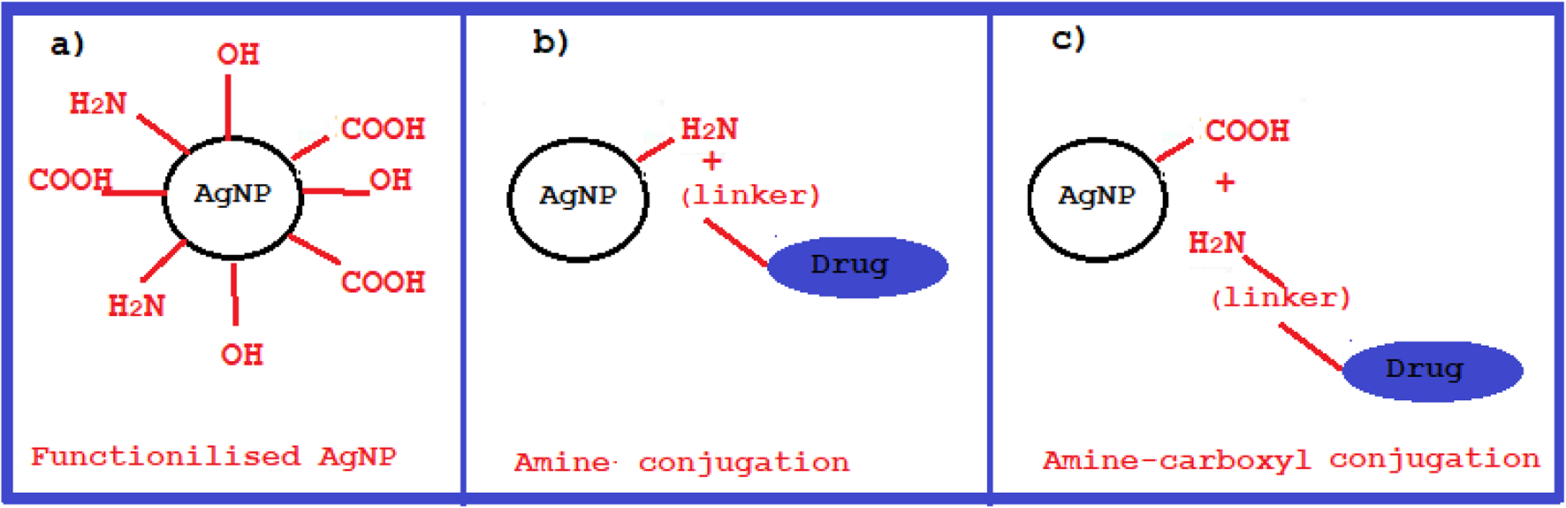
Schematic diagram of toxic drug conjugation strategies on the functionalized nanoparticle surface. (**a**) Schematic illustration of the functionalized AgNP surface. (**b**) Amine conjugation pathway, showcasing the attachment of a toxic drug to the amine functional group on the AgNP surface. (**c**) Amine-carboxyl conjugation pathway, demonstrating the covalent bonding between the functional carboxyl group on the AgNP surface and the linked amine group of the toxic drug.

Furthermore, the functional groups present on the surface of the achieved AgNPs, such as hydroxyl (OH), carboxyl (COO), and amine (−H_2_N) groups (Fig. 2a)^12^, offer a unique opportunity for both physical and chemical conjugation with highly toxic chemotherapy drugs. Extensive literature highlights a variety of strategies for self-attachment and/or conjugation of modified nanoparticles with toxic chemotherapy drugs like duxorubin, CB7-cisplatin, and CB6-nedaplatin, involving hydrogen bonding, covalent bonding, Amine-carboxyl bonding, hydrophobic interactions, electrostatic interactions, π–π stacking, and van der Waals forces^22,31–33^. Consequently, the functionalized AgNPs obtained in our study demonstrate significant potential for physical and chemical conjugation with toxic drugs^32,33^. (Fig. 2b and c).

### Characterization functionalized AgNPs

**Figure 3:**
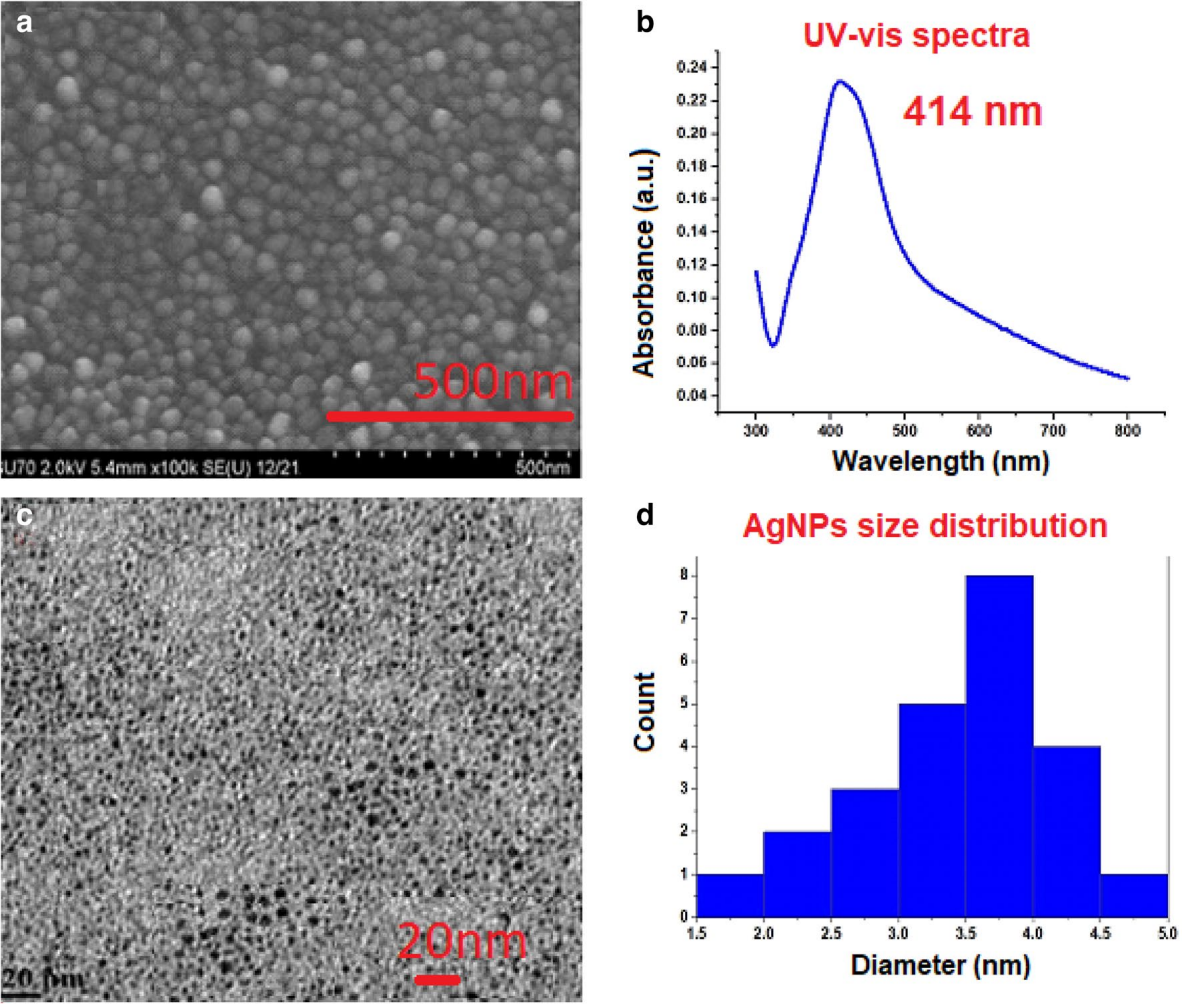
Images (**a**) shows SEM taken at 500 nm scale; (**b**) illustrated UV–vis analysis spectra Fig. 3. (**c**) represent TEM images at 20 nm scale. (**d**) Is histogram where size of the AgNPs shown in range between 1.5 and 5 nm.

First we perform a morphology examination of the AgNP colloidal samples. In order to remedy this, colloidal AgNPs nanocomposite solution was applied with an applicator to a glass surface, spun, and then maintained in a vacuum oven for one night at 20 °C. The surface morphology of the manufactured spin-coated film was assessed using SEM (Fig. 3a). The results show a distinct spontaneous one-to-one AgNPs nucleation in the nanocomposite structure. The unique self-assembled pattern and morphological composition of the AgNPs stabilized in hybrid nanocomposite matrix were revealed by SEM pictures. After this UV–vis analyses was performed on freshly prepared colloidal AgNPs nanocomposite solution (Fig. 3b). It is known that numerous studies have concentrated on noble metals (such as Au and Ag) that show high localized surface plasmon resonance (LSPR) in the visible light domain^13,14,16,54–56^. LSPRs' size, morphology, composition, surface chemistry, and surroundings all affect them in some way. A blue light led exposed AgNPs nanocomposite sample concentration was adjusted to 0.1% and absorption spectra was examined from 300 to 800 nm. Figure 3b shows that at 414 nm, a distinctive LSPR peak develops, denoting the emergence of silver nanoparticles. This strong absorption peak's observation finally proves that the reduction of Ag(I) ions to Ag NPs under the stimulus of blue LED light during the production of AgNP in hybrid metal polymer composition was successful. These findings show that the biocompatible nanocomposite is capable of stabilizing and decreasing AgNPs. The TEM was used to look more closely at the typical size of nanoparticles. For TEM images (Fig. 3c), a fresh hydrogel sample was utilized. The sample concentration was adjusted to approximately 0.01% (w/w). Subsequently, a further dilution of this solution up to 0.005% (w/w) were applied onto the carbon-coated copper grids, followed by overnight evaporation at room temperature. This process resulted in obtaining thinly sliced samples for analysis.. In the examined sample (Fig. 3c), the AgNPs have been uniformly distributed and have an average particle size of about 3.5 nm (Fig. 3d).

Please note that the SEM images primarily illustrate surface morphology, revealing spherical AgNPs encapsulated within a pure carboxylcellulose-based matrix. The SEM analysis (Fig. 3a) suggested an approximate size range of 20–30 nm for the AgNPs, attributing this size estimation to their encapsulation within the 100% pure hydrogel nanocomposite, while the TEM (Fig. 3c)analysis revealed an average size range between of 3–4 nm for the exposed AgNPs.

## Simulation

Nanobubbles have a multi-layered structure that consists of nucleation, air and shell respectively. Then, parameters for each layer can be defined separately such as height, permittivity and permeability^57^. It has been validated that the layers’ heights are adequately shorter than the wavelength, homogeneous and anisotropic multi-layered structure can be seen as whole^57^.

The derived power density per volume (W/m^3^) inter-related Electric Field and wave power^57^

1 $${p}_{l}\left(r,t\right)= \frac{{n}_{1}}{4c}\sqrt{\frac{{\epsilon }^{e}}{{\mu }^{e}} }{\Vert \mathrm{\rm E}(r,t)\Vert }^{2}\frac{1}{r}$$

Note that n_1_ is refractive index of the initial layer, c is the light velocity, **E** is the electric field,*** r*** is the spherical distance to the nucleation, and ${\epsilon }^{e}$; ${\mu }^{e}$ effective dielectric permittivity, and magnetic permeability. The anisotropy caused to the change of the total refractive index $(n=\sqrt{{\epsilon }^{e}{\mu }^{e}})$ as non-linear dispersion. Besides, nonlinear dispersion result in compression and focusing different than the linear dispersion. The temperature field *T*(*r, t*) for 0 ≤ r ≤ L around NPs, with spherical geometry and constant thermal features, is managed by the spherical Fourier heat equation^58^:

2 $${\partial }_{t}T\left(r,t\right)= \frac{{p}_{l}(r,t)}{\rho {c}_{p}}+\frac{\kappa }{{r}^{2}}{\partial }_{r}({r}^{2}{\partial }_{r}T\left(r,t\right))$$

**Figure 4:**
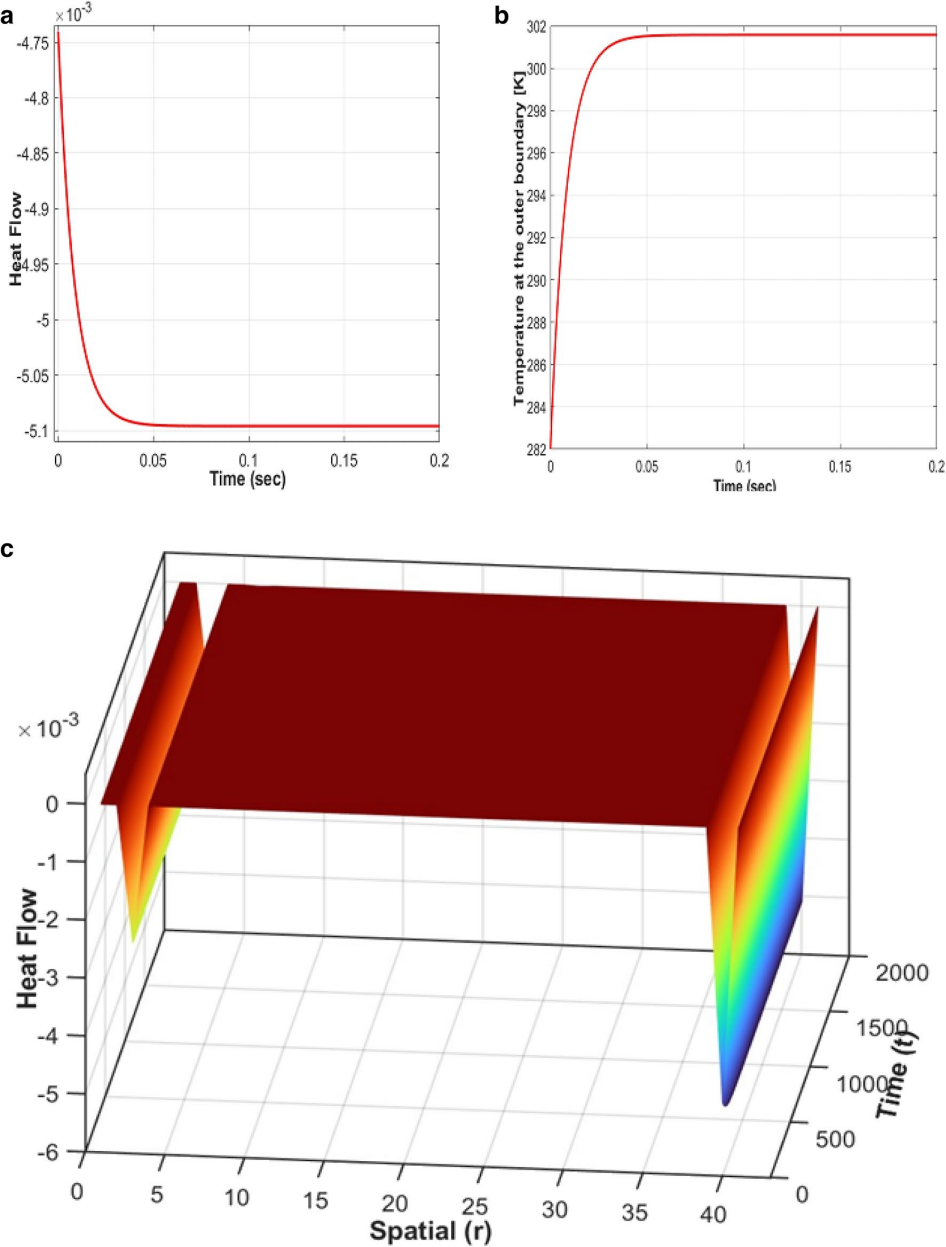
(**a**) Illustrates the Laplacian of the temperature or heat flow. In (**b**), the graph depicts the temperature (in Kelvin) at the outer boundary plotted against time (in seconds) where the laser light initially interacts, at r = 1 mm. Figure 4c represents the 3D Heat Flow visualization, highlighting the boundaries of the layers and the multi-layered structure. The time and spatial coordinates are discretized, with 10,000 time samples (ranging from 0 to 1 s) and 40 spatial samples (1 mm) utilized for the analysis.

Note that $\kappa , \rho$ and c_p_ are thermal diffusivity, density, and heat capacity of liquid, $\kappa \rho$ c_p_ = 1 for water simply, and ***p***_***l***_*** (r, t)*** is the injected power density as above. Approximately adiabatic boundary conditions are given as $\partial$ *rT* (0, *t*) = 0; $\partial$ *rT*(*L, t*) = 0. Steady state solution is obtained for vanishing Temperature gradient^57^,

3 $$T\left(r,t\right)= \frac{A}{r}-Cr {e}^{-\alpha t}+B$$

where A, B, C, and α are constants. Because of absorption and heat conversion, the exponential term will fade out rapidly where C is the control coefficient. Then the first term will be attenuated by distance linearly. Certainly, temperature and pressure will reach the steady state, e.g. see Fig. 4a and b. At the beginning, laser light as controlled source can dominate the production of bubbles and initial stimulation. Upon completion of initial stage, source is turned off, then bubbles stayed steady longer, e.g. see Fig. 4b. Boundary conditions for nucleation and outer surface are validated through the extensive simulations. Thus, we showed that the insulation at the borders is satisfied with sufficiently low temperature gradient. Heat flow changes/absorptions around the boundaries and multi-layered anisotropy are proved by simulations, e.g. see Fig. 4c.

One of our major contributions is to relate the refractive index to temperature gradient. Thus, we can implement the experiment strongly for topological changes. The achieved steady state is satisfactory for long time. Additionally, simulated attenuation constant, α = 0.12 cm^−1^^59^ is chosen in compliance with the susceptibility of AgNPs at measured wavelength, 414 nm.

Thus, attenuation calculation give the idea of the complex part of refractive index inside the bubble that almost matched the experimental result which is *μ*_*a*_ = 0.21 cm^−1^ (absorption). The experimental results validated our model and simulations firmly, e.g. long life-time, pressure balance, compatible with thermal equilibrium at physical boundaries and measurements^57^. In future, improved implementation for advanced experiments will be helpful furthermore.

## Results and discussion

**Figure 5:**
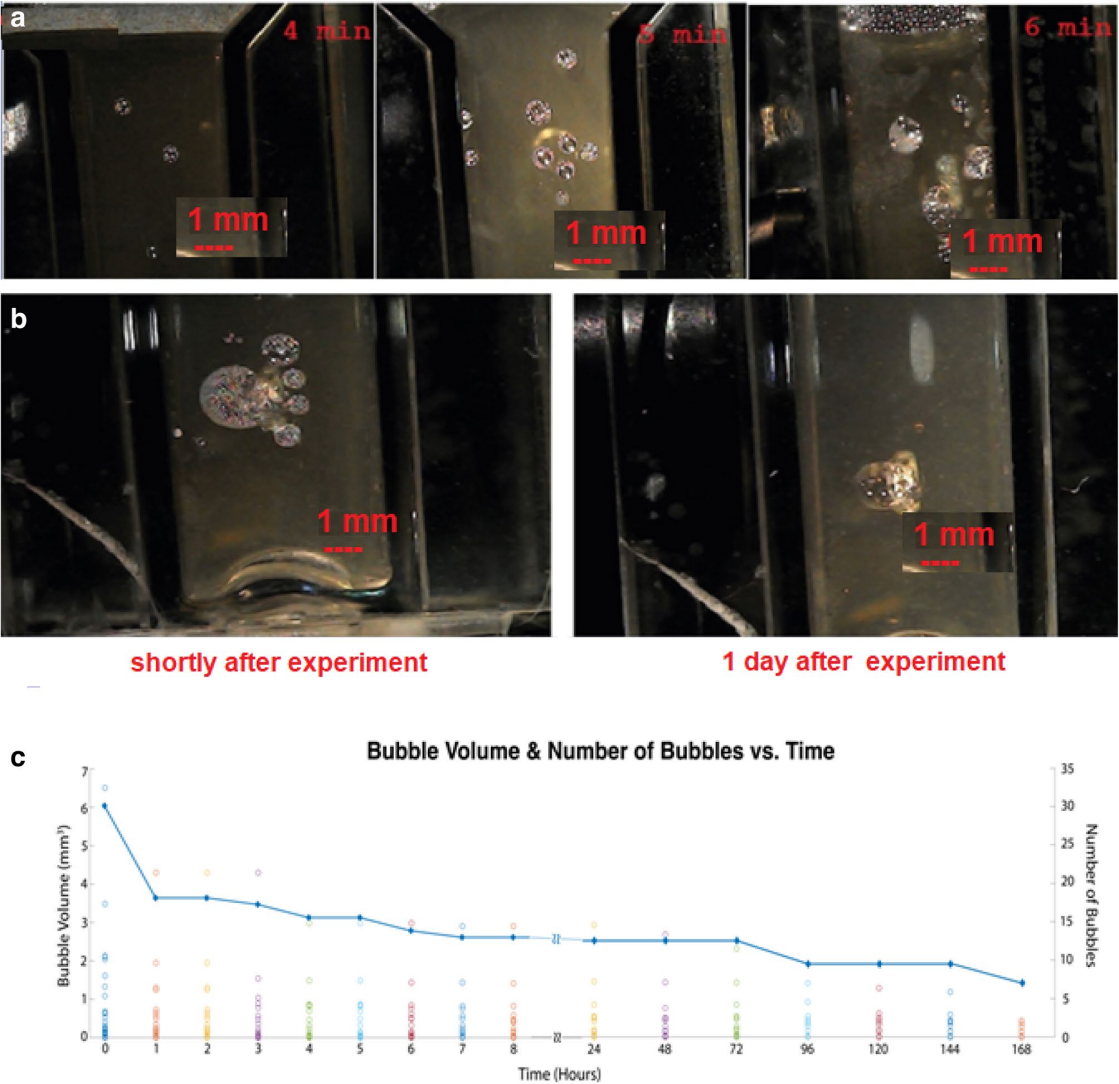
(**a**) Snapshot of bubbles right after the experiments, for three different energy exposures (from left to right 240 J, 300 J, and 360 J.); (**b**) is Snapshot of bubbles, left one right after the experiment, the right one 1 day later; spot size 2 mm; excitation time is 3 min. Figure 5c Bubble size/volume and number of bubbles v. s. time.

Each exposure resulted in the formation of multiple bubbles (Fig. 5a), whose dimensions were calculated using ImageJ software. Figure 5b displays a physical image of bubbles that appeared at the 2 mm spot after 3 min of excitation. The image on the left was captured shortly after the experiment, while the image on the right was taken 24 h later. Figure 5 illustrates the observed bubble diameters that were generated as a result of all 6 experiments, as a function of time. Bubble dimensions were deduced based on CMOS camera images and the use of ImageJ/Fiji software. The dimensions were updated every 12 h to form Fig. 5c, where both total bubble volume as well as number of bubbles are depicted.

Continuous line in the Fug.5c presents the line connecting the number of bubbles data that was acquired every 12 h. It can be considered as an interpolation of the number of bubbles data. Regarding to data points of the experiment this line is now corrected in the revised manuscript to reflect the decrease in number of bubbles in the last time instants progressively.

We initially deduced the extinction coefficient of the nanoparticle solution based on Beer Lambert’s Law. Accordingly we relate impinging input power (P_i_) and put power output that exits the cuvette (P_o_) as;

$${P}_{o}={P}_{i}\left(1-2{\frac{({n}_{g}-{n}_{a})}{{({n}_{g}+{n}_{a})}^{2}}}^{2}\right){e}^{-({\mu }_{a}+{\mu }_{s})L}$$

where *n*_*g*_ is the refractive index of the cuvette, *n*_*a*_ is the refractive index of air, L is the length that light travels inside the cuvette and finally *μ*_*a*_ and* μ*_*s*_ are the absorption and scattering coefficients. We neglect the reflections between the cuvette and the nanoparticle solution (as they have close refractive indexes). We conducted two output vs. input power measurements when the cuvette was filled with distilled water and the nanoparticle solution. The extinction in the distilled power solution is attributed only to absorption phenomena while the extinction in the nanoparticle solution is attributed to both scattering and absorption. We take *n*_*g*_ = 1.5, *n*_*a*_ = 1, L = 1 cm and found the overall transmissions (*P*_*i*_/*P*_*o*_) to be 74.6% and 62.7% for distilled water and nanoparticle solution, respectively. Based on the observed transmission characteristics, and the Beer-Lambert Law we deduce *μ*_*a*_ = 0.21 cm^−1^ absorption) and* μ*_*s*_ = 0.17 cm^−1^ (scattering).

Overall, the experiments reveal that near 40% of the formed bubbles N = 31 (bubble number) lasted for more than a day and near 30% survived as long as a week by optimality of chosen physical parameters.

This link features a video that illustrates the process of bubble nucleation and formation: (see supplementary figures). We have further observed that the bubbles with smaller volume have longer life cycle than the bigger ones. (Fig. 5c).

Note that the bubbles were initially observed for a total laser energy of 240 J for a laser beam area of 12.5 mm^2^, revealing a minimum energy density of 19.2 J/mm^2^ to induce the bubbles. Considering average bubble diameter of 540 μm and an average cross-sectional area of 0.92 mm^2^ at this energy, one can conclude that the bubbles are induced with a minimum of 17.7 Joule of energy on the average.

It is fascinating to observe that the Near-boundary behavior and bubble dynamics, including volume and number, exhibit remarkable similarities with those documented in the^36^. The correlation established between laser power and the mechanism of bubble formation provides compelling evidence that both our experimental and simulation results closely align with the findings presented by Angelskiy at al.^36^. Notably, the laser power level (2 watts) and temperature distribution (curve starting at 28 °C) agree significantly with the data depicted in Fig. 2 of the same paper. These congruent results not only validate our research but also reinforce the robustness and reliability of the previous findings.

In our experiments, we observed that nanobubble shrinks, its radius decreases and the Laplace pressure stemming faster dissolution process refer to video (Supplementary materials). By reaching thermal equilibrium, in a shrinking nanobubble, the radius of curvature increases as opposed to decrease in radius length. Stability of nano bubbles result in shrinkage due to particles on the boundary jam together, generating a growing mechanical stress to overcome Laplace pressure^60^.

Even it is essential to highlight that while the microbubbles observed by Angelskiy et al.^36^, in their studies can reach lifetimes of up to several tens of minutes^36^ or exist for an extended period only under continuous energy "feeding" regimes (0.3W), as opposed in our research, the bubbles obtained by short-duration excitations, achieved an extended lifetime of over seven days, which was intentionally extended by the end of the experiment. This finding demonstrates a notable difference in the longevity of the bubbles between the two studies and underscores the unique characteristics of our biocompatible colloidal solution and experimental conditions.

## Conclusions

We have designed a sophisticated structure for the engineered ultra-diluted biocompatible aqueous nanocomposite system, allowing for the formation of self-excited sub-millimeters bubbles. Through this approach, we achieved long-term stable bubbles and gained control over their lifespan using advanced methods and modeling efforts. By exploiting the structural properties and exclusive synthesis of 3-D hybrid polymer matrices, we were able to attain highly stable bubbles without the need for continuous stimulation. Additionally, the bubble formation model successfully represents the multi-layered and anisotropic colloidal nanocomposite media, incorporating simulated anisotropy and accurately capturing the inherent multi-layered and anisotropic nature of the physical system.

In our experimental study, we established a significant correlation between bubble volume and its lifetime, with smaller bubbles demonstrating longer lifespans compared to larger ones. This finding offers valuable insights for future applications. The experimental and numerical results firmly validated our model and simulations, exhibiting long lifetimes that were consistent with physical boundaries and experimental measurements.

One crucial aspect we explored was the extended lifespan of these MBs, surpassing seven days even when the experiment was intentionally concluded. This remarkable longevity far exceeds what has been reported in existing literature and defies expectations.

The potential applications of these functionalized AgNP-induced MBs are particularly exciting. By easily attaching toxic cancer drugs to AgNPs through electrostatic and/or chemical bonds, these drugs can be safely encapsulated within the bubbles and directed by sound waves can reach deeper into the body. MBs hold promise for targeted administration of toxic cancer drugs, facilitating advanced cancer treatments like chemotherapy, radiotherapy, and drug delivery to highly specific locations within the human body. Such innovative approaches have the potential to revolutionize cancer treatment strategies, offering safer and more effective therapeutic options in the future.

The number of bubbles can be adjusted depending on the clinical application and required dosages. Yet, it is noteworthy to mention that the number of bubbles can be increased upon applying multiple laser exposures at different locations. This could be achieved through the placement of the laser on a 2-dimensional translation (motorized or manual) stage or through steering the beam on the sample using scanning mirrors such as galvanometric or microelectromechanical-system (MEMS) based miniaturized scanners.

## Materials and methods

Carboxymethyl cellulose sodium salt, is the main polymer (in text mentioned as CMC) with average Mw ~ 90,000; Octadecylamine ≥ 99% (GC) is a surfactant (is mentioned as ODA); Poly (acrylic acid-co-maleic acid) with Mw = 3000, 50 wt% in H2O, is a reactive copolymer, mentioned in the text as MA, and silver nitrate (AgNO3, 99.995%, d = 4.35 g cm^−3^) have obtained from Sigma-Aldrich. Distilled water was used as a solvent. All obtained chemicals were used without further purification.

### Syntheses procedure

AgNPs in the colloidal blend were found using blue LED source. The LED syntheses protocol was previously described in our study^12^. Briefly blend of hybrid colloidal with silver salts was stirred up to a homogenous state and then transferred into a quartz cuvette. After this, the cuvette containing 1 mL of nanocomposite was stimulated by blue LED for 15–20 min.

### Characterization techniques

Field Emission Scanning Electron Microscope (FESEM)—Hitachi SU70 with an image resolution of approximately 1 nm was utilized for obtaining samples surface images. High-Resolution Transmission Electron Microscopy (HRTEM JEOL JEM 2010, a high-resolution electron microscope equipped with an accelerating voltage of 200 kV and a point resolution of 0.19 nm) was employed for capturing TEM images to investigate the internal morphology, nano-sizes, and diameter distributions. For UV–vis measurements, a Spectrophotometer UV5 with a range of 190–1100 nm was employed.

### Optical experiments

All optical experiments in this study were conducted at the Faculty of Electrical-Electronics Engineering, Istanbul Technical University, Istanbul, Turkey, employing a specifically designed custom-built low-power laser setup.

### Supplementary Information

Supplementary Video 1.
